# Motion Planning for a Legged Robot with Dynamic Characteristics

**DOI:** 10.3390/s24186070

**Published:** 2024-09-19

**Authors:** Xu Liu, Limin Yang, Zhijun Chen, Jiangwei Zhong, Feng Gao

**Affiliations:** 1 State Key Laboratory of Mechanical System and Vibration, School of Mechanical Engineering, Shanghai Jiao Tong University, Shanghai 200240, China; xu.liu@sjtu.edu.cn (X.L.); ylm20159@sjtu.edu.cn (L.Y.); ppq67hwuxe@sjtu.edu.cn (Z.C.); 2 The Lenovo Corporation, Beijing 100085, China; zhongjw@lenovo.com

**Keywords:** legged soccer robot, motion planning, dynamic soccer skill, gait-cycle planning, gait scheduler, leg controller

## Abstract

Legged soccer robots present a significant challenge in robotics owing to the need for seamless integration of perception, manipulation, and dynamic movement. While existing models often depend on external perception or static techniques, our study aims to develop a robot with dynamic and untethered capabilities. We have introduced a motion planner that allows the robot to excel in dynamic shooting and dribbling. Initially, it identifies and predicts the position of the ball using a rolling model. The robot then pursues the ball, using a novel optimization-based cycle planner, continuously adjusting its gait cycle. This enables the robot to kick without stopping its forward motion near the ball. Each leg is assigned a specific role (stance, swing, pre-kick, or kick), as determined by a gait scheduler. Different leg controllers were used for tailored tiptoe trajectory planning and control. We validated our approach using real-world penalty shot experiments (5 out of 12 successful), cycle adjustment tests (11 out of 12 successful), and dynamic dribbling assessments. The results demonstrate that legged robots can overcome onboard capability limitations and achieve dynamic mobility and manipulation.

## 1. Introduction

Soccer is recognized as a benchmark challenge in robotics, offering a comprehensive testbed for evaluating the capabilities of robotic controllers in complex environments, multifaceted tasks, and highly dynamic scenarios. Early RoboCup competitions are well known for their adoption of rule-based strategies, covering aspects such as kicking, passing, and shooting techniques for quadruped [1] and bipedal robots [2]. These approaches typically depend on the invocation and combination of various motion primitives to achieve victory. Subsequent research has focused on enhancing the specific skills of soccer robots, including shooting [3], dribbling [4,5], and juggling [6] in simulations, along with real-world precision shooting [7], goal defense [8], static dribbling [9], and circus ball challenge [10]. Notably, algorithms validated on physical robots often depend on external devices for assistance, such as stationary cameras or motion-capture systems. The motion of the sensory components, ball, or robot increases the uncertainty of the system, complicating the interaction between the robot and the ball. Through reinforcement learning, a study [11] achieved the first simulated humanoid robot kick without stopping its forward motion, while another study [12] demonstrated, in reality, dynamic dribbling without the need for tethers or external sensing.

The highly dynamic soccer skills of legged robots represent a set of complex tasks that require a tight integration of visual perception, object manipulation, and dynamic movement [12]. Firstly, visual perception is critical. Accurately determining the soccer ball’s position is essential for robot–ball interaction. The most reliable method is using external motion-capture systems, which eliminate difficulties in ball recognition across various backgrounds and provide highly precise positional data. Some studies employ externally fixed cameras, but ensuring stable recognition and dealing with inherent accuracy limitations is problematic. Mounting cameras onto legged robots introduces additional complexities due to body sway and onboard computational limitations, leading to potential issues, like low frame rates, target loss, and poor positional accuracy. Secondly, object manipulation on a floating base is complex. Legged robots constantly adjust their posture, and various internal and external forces affect their stability. Performing precise manipulations on such an unstable platform adds another layer of difficulty. Body sway can inadvertently move the kicking leg, and the vigorous motion of kicking can further destabilize the robot. Thirdly, the dynamic motion of legged robots requires precise timing. As the robot approaches the ball, it must position a leg whose workspace includes the necessary kicking trajectory, and this leg must be in the swing phase at the exact moment. Unlike most existing studies where robots walk to the ball, stop, stabilize, and then kick, our approach solves the problem of timing, balancing body motion while simultaneously manipulating the ball, rather than relying on static kicking after halting movement. Finally, the sensitivity of the kicking action means that even slight deviations in kicking position can lead to significant misdirection of the ball.

### 1.1. Related Work

**Visual perception**: The categorization can be divided into indoor installations [7,8] and onboard systems [12] based on the mounting location of the camera; onboard scenarios deteriorate the image quality. Visual systems are classified into monocular setups [13], binocular cameras [7,8], and multi-camera arrays in motion-capture systems [9,10] depending on the number of cameras used. Detection techniques include conventional vision-processing methods, such as thresholding [14] equalized color-based clustering [13]; circular gradient methods [15]; and deep-learning approaches, such as YOLO [16].

**Object manipulation**: In legged soccer robots, kicking actions can be conceptualized as an object-manipulation problem using a floating-base manipulator. Research in manipulation has achieved extensive success using fixed-base or fully actuated mobile-based manipulators. This includes tasks such as hitting [17], catching [14,18], and throwing [19]. However, less exploration has been performed in cases involving floating bases. The study by [20] utilizes multi-task reinforcement learning to achieve dynamic object tracking and grasping with a manipulator mounted on a wheeled platform. A whole-body planning framework that uses a quadrupedal mobile manipulator to push or pull a heavy resistive door was introduced in a study [21]. The study by [22] proposes a disturbance prediction control framework that enables versatile mobile manipulation using a quadrupedal mobile arm. Another study [23] integrated a learning-based locomotion policy with a model-based manipulator for mobile legged manipulators. The stability control and uncertainty in a floating base make precise object manipulation challenging. As a result, a study [10] presented a quadrupedal robot that performs the circus-ball challenge while lying on its back, effectively circumventing the influence of a floating base.

When the object being manipulated, such as a soccer ball, has significant mobility, incorporating a trajectory model can potentially enhance the success rate of the operation. An example of this is a study [14] that uses an extended Kalman filter based on Newtonian mechanics to determine the ball’s three-dimensional position. Additionally, the interaction between a ball, which is an imperfectly elastic body, and a mechanical arm or environment introduces further unpredictability into its state. The dynamics of a typical ball bounce are described in [24]. Another study [25] includes a model related to the changes in flight and spin velocity before and after bouncing. The aerodynamic effects of lift and drag on the trajectory were investigated in [17].

**Dynamic movement**: In the study of dynamic agility of legged robots, several whole-body optimization and control algorithms based on dynamic models have achieved widespread success. These include strategies based on simplified dynamics, such as Centroid Balance Control (CBC) [26]; whole-body dynamics, such as Whole Body Control; and future-state prediction methods, such as Model Predictive Control [27]. Recently, model-free reinforcement learning approaches have also seen considerable success in enhancing the agility control of legged robots. Both zero-shot transfer [28] and methods that incorporate real-world data [29] have shown potential in bridging the gap between simulations and reality to some extent.

Success has been achieved in the distinct domains of visual perception, object manipulation, and dynamic movement. However, tightly integrating the State-of-the-Art methods across these diverse fields poses a substantial challenge. To circumvent complex modelling and planning issues while fully exploring the potential of robotic movements, one study [7] employed hierarchical reinforcement learning to develop precise soccer shooting skills for a quadruped robot. However, in this case, the soccer robot remained stationary while kicking the ball and required an external depth camera. In contrast, another study [12] leveraged reinforcement learning to achieve dribbling while moving using only an onboard camera rather than a static kick [9] or pushing the ball with its body. Beyond these, few studies have successfully demonstrated highly dynamic untethered soccer skills with legged robots. Recently, the study by [30] employed deep reinforcement learning to realize more adversarial kicking behaviors on bipedal robots, encompassing actions such as defense and offense, as well as game strategies. However, it still requires a motion capture system to provide the positions of the ball and the robot.

### 1.2. Contributions

In this study, we overcame computational limitations and motion blur to achieve onboard detection, localization, and tracking of the soccer ball, freeing the robot from dependence on external sensors. We proposed a novel rule-based motion planner that simultaneously optimizes the locomotion and manipulation behaviors of the legged robot, enabling dynamic soccer ball manipulation and movement. After perceiving and predicting the ball position using an onboard camera with a rolling model, the robot is instructed to chase the ball while avoiding obstacles. Subsequently, the corresponding planning of the stance and swing legs is performed in the motion control computer. As the ball approaches the zone for kicking, a planning module of the kicking leg issues tiptoe trajectory commands for the kick. To ensure the dynamic nature of the kick, an optimization-based cycle-planning module is introduced to continually adjust the gait cycle, enabling the kick while moving. We have developed a legged soccer robot capable of mastering dynamic shooting and dribbling skills. It can be used to manipulate a soccer ball on surfaces with varying friction and maintain balance during movement. Based on mathematical models and clear rules, our soccer robot shows promise for stable and robust performance. A series of real-world penalty shot experiments (with a success rate of 5 out of 12), cycle adjustment experiments (with a success rate of 11 out of 12), and dynamic dribbling tests further demonstrated the effectiveness of our proposed algorithm.

The contributions and novelty of this work include the following:

- By combining the fine-tuned YOLO weights, RANSAC method, and ball-rolling model, a new use case and approach are provided for the detection, localization, and tracking of the ball under onboard constraints and motion blur.
- A new gait scheduler was proposed based on the phase and workspace of each leg, allowing the legs to flexibly switch between the swing, stance, pre-kick, and kicking states.
- A new leg-trajectory planning in the kicking state was proposed.
- A novel gait cycle-planning method was proposed which ensures the robot’s dynamic kicking by continually optimizing its gait cycle.

## 2. Materials

**Figure 1:**
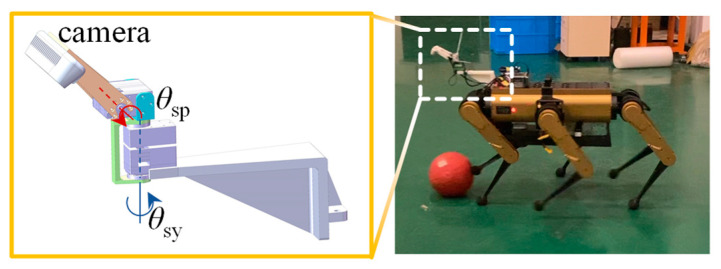
Hexapod robot for kicking ball.

All experiments were conducted using a self-developed hexapod robot and a size 4 soccer ball. In its compact state, the hexapod robot can be accommodated within a box measuring 780 mm × 600 mm × 300 mm, while it extends up to 500 mm in height when in its standing posture. As depicted in Figure 1, each leg of the hexapod robot is equipped with three degrees of freedom (hip, thigh, and shank), allowing for the acquisition of joint position, speed, and force information through joint encoders and actuator currents.

**Figure 2:**
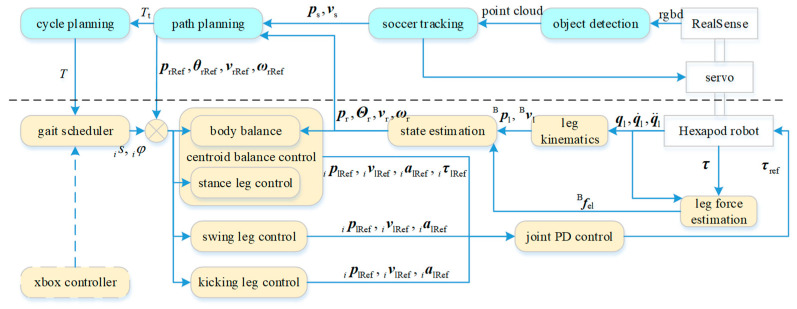
Control-system block diagram. Components above the dotted line are executed on the upper computer, whereas those below the dotted line run on the lower computer. Within this framework, the modules represented by the blue boxes operate at a frequency of 15 Hz, and the modules within the yellow boxes function at 1 kHz.

Figure 2 illustrates that the joint information, after being processed through kinematic and dynamic calculations, along with data from the inertial measurement unit, is inputted into a state-estimation module. This module accurately estimates the state of the robot and serves as the foundation for the motion control. In addition to state estimation, other modules related to motion control, such as the gait scheduler, body balance, and leg control, are executed on the embedded motion control computer, DFI WL051, at a frequency of 1 kHz.

More advanced functionalities, such as object detection and tracking, path planning, and gait-cycle planning, are executed on an onboard NVIDIA Jetson Xavier NX unit. Object detection and tracking are based on images captured using a RealSense D435i depth camera. Given that the camera’s RGB field of view is only 69° × 42°, which does not suffice to cover both the nearby (especially the area directly beneath) and adjacent areas, an additional two-degree-of-freedom servo unit is utilized to hold the camera, ensuring that it remains oriented toward the soccer ball. The path-planning and gait cycle-planning modules manage information from the robot’s states and vision, generating high-level control commands at a frequency of 15 Hz.

## 3. Methods

The control-system block diagram shown in Figure 2 encapsulates a comprehensive array of functional modules required for dynamic soccer skills by a legged robot, spanning from perception to decision-making, and from body control to object manipulation. These modules will be sequentially introduced in the following subsections. Section 3.1 discusses the detection and tracking related to the soccer balls. Based on the current state information of the ball and robot (primarily the pose and velocity), the path-planning generates the target state for the robot body at the next moment. Section 3.2 presents a gait scheduler that elucidates the selection of the stance, swing, and kicking legs. Section 3.3 introduces the control of the stance legs, as well as the generation of tiptoe trajectories for the swing and kicking legs. Finally, to enhance the dynamic characteristics of the kick, cycle planning is introduced in Section 3.4.

### 3.1. Object Detection and Tracking

#### 3.1.1. Fine-Tuned YOLOv5 Model Weights

We adopted YOLOv5 model weights, which were pre-trained on the dataset [16], and fine-tuned them using 120 hand-labelled color images. This module, leveraging CUDA acceleration, can process images at a resolution of 1280 × 720 pixels and a throughput of 15 frames per second.

**Figure 3:**
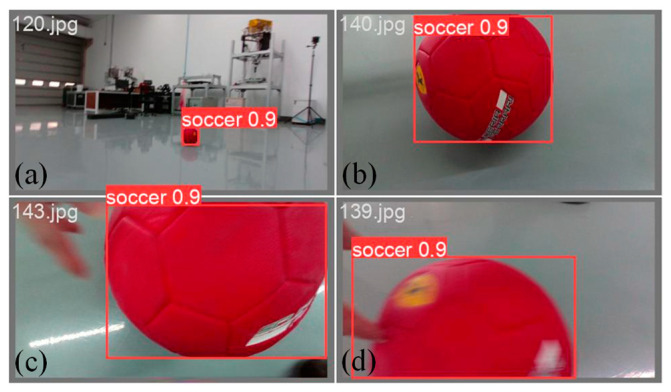
Detection of the ball achieves a confidence level of 90% under four slightly adverse conditions: (**a**) ball is far from the camera (5 m), (**b**) ball is close to the camera, (**c**) only a part of the ball is seen, and (**d**) motion blur.

Although the pre-trained YOLOv5 model can detect ball-like objects, two substantial issues were observed. First, the detection rate significantly dropped when no humans were near the ball. This is likely due to the training dataset being composed of sports activities involving human participation. The second issue arose when the ball was close to the camera and occupied a substantial portion of the frame, leading to a drastic decrease in the detection rate. We addressed these issues by collecting and manually labelling 120 images, both with and without humans, and with the ball at varying distances. The results are shown in Figure 3.

#### 3.1.2. Sphere Fitting

**Figure 4:**
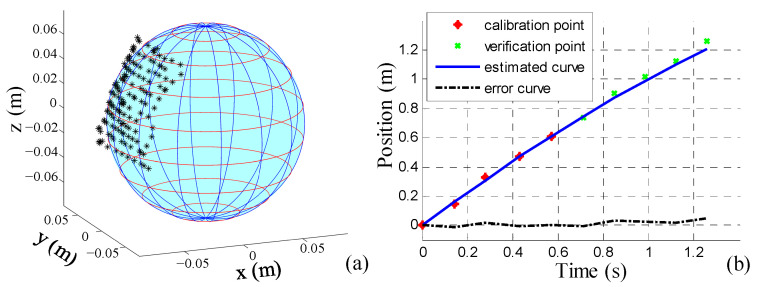
(**a**) RANSAC method is employed to fit the selected 144 points around the center of the detected object to a spherical surface. (**b**) Ball’s velocity is estimated from the continuously acquired data on the center of the ball, and the subsequent positions of the soccer ball over time are predicted. The maximum error noted in the figure is 0.047 m.

To obtain accurate coordinates for the center of the ball, we used the coordinates of 144 points near the center of the ball object detected by YOLO to fit a spherical surface, as shown in Figure 4a. Given the significant noise in depth information, we employed the RANSAC method [31] to eliminate gross errors. A detection is considered valid only if the fitted radius differs only slightly from the actual radius, *r*_s_, of the ball.

We used a two-degree-of-freedom servo unit to reorient the camera, ensuring that the ball remained at the center of the image. We adopted PD control with a dead zone to prevent frequent adjustments and servo jitter.

#### 3.1.3. Identification of the Rolling Friction Coefficient

A soccer ball rolling solely on the ground gradually decelerates owing to the friction force. Because the ball did not bounce vertically, its potential energy remained unchanged. The kinetic energy, *T*_s_, of the ball comprises translational and rotational energies:

(1) $$T_{s}=\frac{1}{2}m_{s}v_{s}^{2}+\frac{1}{2}J_{s}\omega_{s}^{2}=\frac{1}{2}\left(m_{s}+\frac{J_{s}}{{r_{s}}^{2}}\right)v_{s}^{2}$$

where *m*_s_ represents the mass of the ball, *J*_s_ is the moment of the inertia, and *v*_s_ and *ω*_s_ are the scalar forms of the linear and angular velocities, respectively. The generalized force, *f*_s_, acting on the ball comprises Coulomb friction, viscous friction, and air resistance [14], and it can be expressed as follows:

(2) $$f_{s}=-f_{\mathrm{sc}}-f_{\mathrm{sv}}v_{s}-f_{\mathrm{sd}}v_{s}^{2}$$

where *f*_sc_, *f*_sv_, and *f*_sd_ represent the Coulomb friction force, viscosity coefficient, and air-resistance coefficient, respectively. Substituting Equations (1) and (2) into the Lagrange equation, $\frac{d}{dt}\left(\frac{\partial L}{\partial \dot{q}}\right)-\frac{\partial L}{\partial q}=f_{s}$, we obtain the following:

(3) $$a_{s}=\frac{-f_{\mathrm{sc}}-f_{\mathrm{sv}}v_{s}-f_{\mathrm{sd}}v_{s}^{2}}{m_{s}+J_{s}/r_{s}^{2}}$$

To prevent the ball from being pushed by friction when stationary in the numerical calculations [32], we use the following:

(4) $$f_{\mathrm{sc}}=\{\begin{array}{c} \begin{array}{cc} 0, & v_{s}=0 \end{array}\\ \begin{array}{cc} v_{s}/k_{s}, & 0<v_{s}\leq k_{s} \end{array}\\ \begin{array}{cc} mg\mu_{s}, & k_{s}<v_{s} \end{array} \end{array}$$

where *μ*_s_ is the sliding friction coefficient. By setting *k*_s_ = 0, Equation (4) is reduced to the classical Coulomb friction model. Considering that a ball undergoing pure rolling on the ground often has a low speed, the air-resistance term in the acceleration is neglected. Integrating the equation twice yields the following:

(5) $$\begin{array}{l} p_{s}\left(t\right)=-\frac{f_{\mathrm{sc}}}{f_{\mathrm{sv}}}\left(t-t_{0}\right)+\frac{\frac{f_{\mathrm{sc}}}{f_{\mathrm{sv}}}+v_{s0}}{\frac{f_{\mathrm{sv}}}{m_{s}+J_{s}/r_{s}^{2}}}\left(1-e^{-\frac{f_{\mathrm{sv}}}{m_{s}+J_{s}/r_{s}^{2}}\left(t-t_{0}\right)}\right)+p_{s0}\\ =c_{s1}\left(t-t_{0}\right)+\frac{-c_{s1}+v_{s0}}{c_{s2}}\left(1-e^{-c_{s2}\left(t-t_{0}\right)}\right)+p_{s0} \end{array}$$

where $c_{s1}=-\frac{f_{\mathrm{sc}}}{f_{\mathrm{sv}}},\;c_{s2}=\frac{f_{\mathrm{sv}}}{m_{s}+J_{s}/r_{s}^{2}}$. In experiments across different terrains, we employed the Gaussian–Newton methods for the sliding-window estimation of these two coefficients, and the ball’s initial velocity, *v*_s0_. As shown in Figure 4b, we identified the coefficients by capturing five consecutive ball positions and their corresponding moments, facilitating the prediction of the ball’s subsequent position, ***p***_s_, and velocity, ***v***_s_, in the world coordinate system.

#### 3.1.4. Path Planning and Chasing Time Prediction

After acquiring the ball position, generating a new path is essential to drive the robot to chase the ball. We used the hybrid A * algorithm proposed in [33] to incorporate potential field boundaries and obstacles, including other robots, to plan a collision-free, smooth trajectory. Moreover, as the robot moves along the planned path, we need to estimate the time required for the robot to reach the zone allowed for the kick. This estimation is based on the path length and the robot’s velocity, and is used in gait-cycle planning in Section 3.4.

### 3.2. Gait Scheduler

#### 3.2.1. Tripod Gait

**Figure 5:**
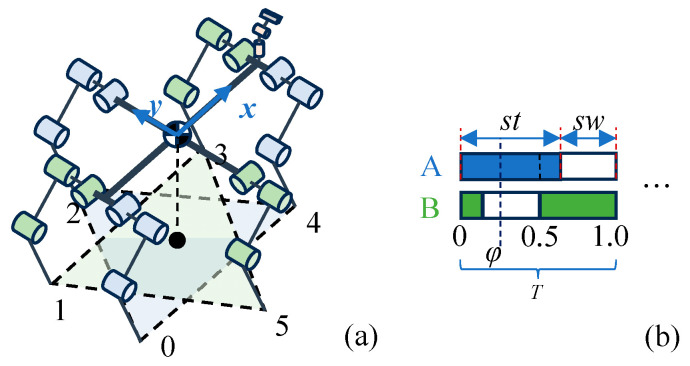
(**a**) Triangle support. (**b**) Duty factor and phase.

In this study, the hexapod robot employs a tripod gait, as depicted in Figure 5a. All legs are divided into two groups: *A* and *B*. Group *A* includes legs numbered 0, 2, and 4, while the remaining legs constitute Group *B*. The robot achieves motion and balance by periodically alternating the stance and swing states between the two groups. Structurally, the robot’s body is symmetrical, with the distance of leg 2 from the x-axis being twice that of legs 0 and 4. This design ensures an even load distribution across all legs, which is advantageous for the implementation of a tripod gait.

The transition between the stance and swing states is determined by the phase, *φ*. The gait cycle, *T*, is defined as the time taken for a complete stance state and swing state by one group of legs. The duty factor, *η*, is defined as the proportion of the cycle occupied by the stance state time, *T*_st_, that is

(6) $$\eta =T_{\mathrm{st}}/T$$

Phase *φ* is defined as the proportion of time elapsed in the current cycle relative to the total cycle time, which is a normalization of time within the cycle. To facilitate the alternating movements of the two groups of legs, phase offsets, *φ*_Ao_ and *φ*_Bo_, are introduced. Consequently, the actual phases of the two groups of legs are given by the following:

(7) $$\begin{array}{l} \varphi_{A}=\varphi +\varphi_{\mathrm{Ao}}\\ \varphi_{B}=\varphi +\varphi_{\mathrm{Bo}} \end{array}$$

The *i*-th leg’s phase, *_i_φ*, is one of the two. When *_i_φ* ∈ 𝒫_st_ = [0, *η*), the leg is in the stance state; when *_i_φ* ∈ 𝒫_sw_ = [*η*, 1), the leg is in the swing state. For the tripod gait, a duty factor, *η*, of 0.5 is common, with *φ*_Ao_ = 0 and *φ*_Bo_ = 0.5. As shown in Figure 5b, in this study, *η* is slightly increased to 0.55. This adjustment serves two purposes: First, it ensures a smooth transition between the stance and swing states, considering the elasticity of the tiptoes. It is crucial to ensure that the new stance legs are fully grounded before lifting the previous stance legs. Second, it facilitates adjustments to the gait cycle and phase offsets during moments when all legs touch the ground. Section 3.4 discusses how altering the cycle and phase can adjust the transition between the stance and swing states, thus enhancing the dynamic characteristics for kicking the ball.

#### 3.2.2. Selection of Kickable Leg

**Figure 6:**
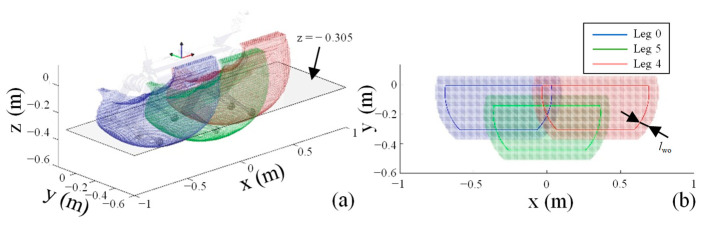
(**a**) Workspace of the three legs on the right side of the body, considering joint constraints. (**b**) Workspace slice at plane z = −0.305 m in the body coordinate system.

Considering the robot’s left–right symmetry, only the workspace of the right three legs was calculated, as shown in Figure 6a, with a specific overlap between them. With the robot’s body center at 0.4 m above the ground and the ball’s radius at 0.095 m, a plane parallel to the ground and 0.305 m from the body center is used to intersect the workspace, as depicted in Figure 6b. The workspace slice for a single leg is a part of a disc, as summarized in Table 1. To avoid reduced mobility near the workspace boundary and to prevent the planned kicking trajectory from deviating significantly from the normal swing trajectory, the workspace slice outline in Figure 6b is inwardly offset by *l*_wo_, serving as the feasible kickable range, *_i_*𝒵_k_, for each leg.

When the distance between the ball and the robot falls below a threshold, the potential kicking leg is determined based on Table 1 and *l*_wo_. When the center of the ball is located within the zone for kicking of any leg, that leg switches to the pre-kick state. If more than one leg enters this pre-kick state, the two nearest legs are designated as the optimal and suboptimal pre-kick legs based on the distance from the ball to each leg. The kicking action is activated if any pre-kick leg is within the corresponding feasible phase range, 𝒫_k_, for the kick; otherwise, they continue to perform their respective swing or stance actions, as indicated by

(8) 𝒫k={[η,φthopt),optimal pre-kick leg     [η,φthsub),suboptimal pre-kick leg

The range 𝒫_k_ ⫋ 𝒫_sw_, and the thresholds _opt_*φ*_th_ and _sub_*φ*_th_ represent the upper limits allowing a leg to switch to the kicking state. As the robot operates in a tripod gait, the optimal and suboptimal pre-kick legs cannot be in the swing state simultaneously, thereby preventing both from being simultaneously activated for a kick. Additionally, _opt_*φ*_th_ > _sub_*φ*_th_ typically because the optimal pre-kick leg is closer to the ball, requiring a slightly smaller margin for the timing of the action. Considering the time prediction error of path planning and the limitations of gait-cycle adjustments, in some cases, when the ball reaches the kickable zone, neither the optimal nor the suboptimal leg is in a feasible phase range. In such situations, the kicking action is delayed until any leg reaches a feasible phase for the kick.

**Figure 7:**
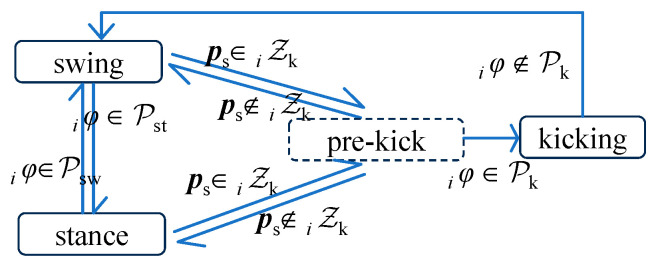
Finite-state machine for legs.

For the state *_i_s* of any leg, there are four possible states: swing, stance, kicking, and pre-kick. The scheduling of these states is summarized in the state machine, as illustrated in Figure 7. In the swing or stance state, when the ball’s position, ***p***_s_, reaches the kickable zone, *_i_*𝒵_k_, the leg switches to the pre-kick state. The controller generates a trajectory for kicking the ball with a tiptoe based on the continuously updated position of the ball. When the phase of the leg in the pre-kick state meets the feasible phase range, 𝒫_k_, the pre-kick leg switches to the kicking state to perform the kicking action.

### 3.3. Leg Planning

#### 3.3.1. Stance-Leg Planning

**Figure 8:**
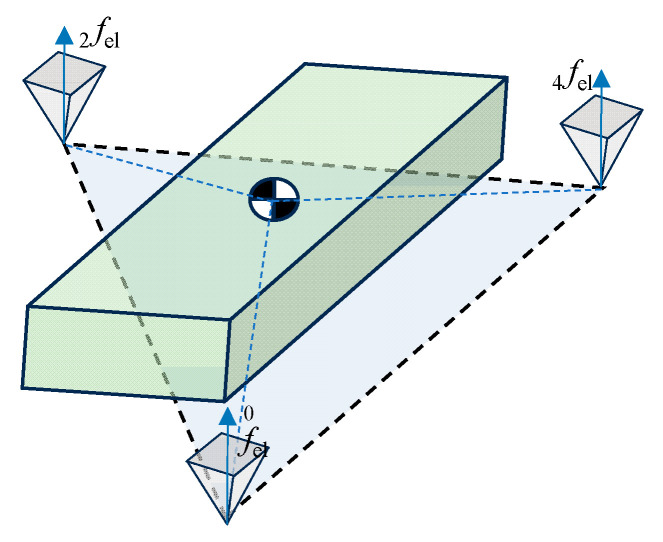
Principle of CBC for the single rigid body.

The locomotion of legged robots depends on the ground reaction forces applied by their stance legs. Owing to the periodic transition between stance and swing states and the constraints of tiptoe–ground friction, a constrained optimization solver is required to distribute the ground reaction forces, ensuring the robot’s balance. Considering that the drive modules of the robot’s legs are located at the hip and the mass of the leg links constitutes only a minor portion of the mass of the entire robot, it is reasonable to allocate the ground reaction forces to the stance legs using the CBC method [34]. To simplify the constraints, the friction cone is replaced with a quadrangular pyramid, as shown in Figure 8. The optimized ground reaction forces then propel the simplified rigid body of the robot to follow the planned states while maintaining balance.

#### 3.3.2. Swing-Leg Planning

Unlike the tiptoes of the stance legs, which remain grounded, the tiptoes of the swing and kicking legs must follow a specified spatial trajectory.

The landing point is the endpoint of the swing leg’s tiptoe trajectory. This study adopts the method presented in [27], which calculates the position of each hip at the next ground contact event. The landing point of the swing leg’s tiptoe is set as half the step length ahead of the hip at the moment of ground contact.

After determining the landing point of the swing leg, the problem of tiptoe trajectory planning becomes a point-to-point planning problem between the lift-off and landing points, subject to a specific step height and boundary constraints. In this study, the tiptoe trajectory in the x and y directions was modelled using a cubic polynomial curve, while the z-direction trajectory, which initially rises and then falls, was formed by joining two cubic polynomial curves.

#### 3.3.3. Kicking-Leg Planning

When considering only the tiptoe trajectory, the kicking leg can be viewed as a special case of the swing leg. The distinction lies in the tiptoe trajectory of the kicking leg in the x, y, and z directions, each consisting of three cubic polynomial curves. As described in Section 3.2, a leg can only activate the kicking state within a specific phase range. When a leg first transitions to the kicking state, its tiptoe position and velocity are recorded as *_i_**p***_l0_ and *_i_**v***_l0_, respectively, and the instant is recorded as *t*_k0_, marking the kicking start point. The expected instant, *t*_k3_; position, *_i_**p***_l3_; and velocity, *_i_**v***_l3_, of the next ground contact event are also recorded if the leg continued its original swing trajectory.

**Figure 9:**
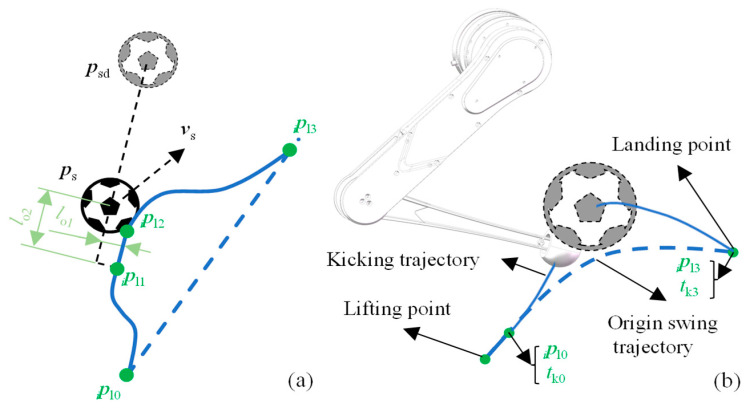
(**a**) Top view schematic of kicking trajectory. (**b**) Spatial schematic of kicking trajectory.

As illustrated in Figure 9, the desired position of the ball is denoted by ***p***_sd_. To kick the ball toward the desired location, the segment pl1ipl2i→ in the kicking trajectory should be parallel to segment $\vec{p_{s}p_{\mathrm{sd}}}$, and the velocity of this segment is proportional to the ball’s target displacement with coefficient *λ*_k1_, that is,

(9) $$v_{l1}=v_{l2}=\lambda_{k1}\vec{p_{s}p_{\mathrm{sd}}}$$

Furthermore, to counteract the velocity of the ball perpendicular to the desired direction, an offset, *l*_o1_, is applied. The offset is proportional to the velocities in the ratio *λ*_k2_:

(10) $$l_{o1}=\lambda_{k2}\frac{v_{s}\times v_{l1}}{{\left\|v_{l1}\right\|}^{2}}$$

To ensure that the tiptoe of the kicking leg does not make premature contact with the ball, the offset *l*_o2_ should satisfy the following condition:

(11) $$l_{o2}>r_{s}+r_{l}$$

where *r*_l_ denotes the radius of the tiptoe. The final trajectory of the leg is obtained by connecting points *_i_**p***_l0_, *_i_**p***_l1_, *_i_**p***_l2_, and *_i_**p***_l3_ using three cubic polynomial curves.

### 3.4. Gait-Cycle Planning

The objective is for the robot to kick the ball directly with its leg in the swing state, immediately upon reaching the ball. This eliminates the need for the robot to stop and lift its leg to kick, as required in static kicking methods. Ideally, when the robot reaches the kicking position, the leg designated to kick the ball should have just entered the swing state. This provides sufficient time for the kicking action and subsequent landing action. Without loss of generality, we assume that the target kicking leg belongs to Group *A*.

**Figure 10:**
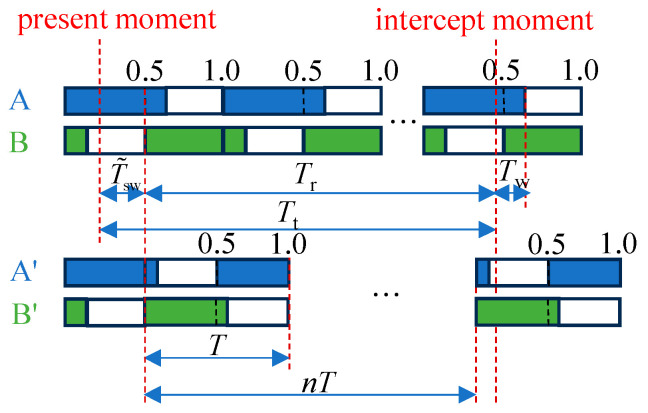
Gait cycle adjustment and phase change in tripod gait. During the process of the robot chasing the ball, the moment when the ball enters the kickable area is defined as the interception moment. Simultaneously, at least one leg enters a pre-kick state.

Determining the total time, *T*_t_, taken by the robot to move from its current position to the interception point is possible owing to path planning. As shown in Figure 10, by adjusting the robot’s gait cycle, *T*, the leg designated for the kick enters the pre-kick state precisely at the beginning of its swing state, eliminating the need for additional waiting time, *T*_w_. To ensure smooth operation of the robot, changes to the gait cycle, *T,* are made only during intervals when all legs are grounded. Thus, the actual available time for adjustment is the remaining time:

(12) Tr=Tt−max{T˜swi},i∈0,1,…,5

where T˜swi is the remaining time for the *i-*th leg to be in the swing state. If the leg is in the stance state, then T˜swi=0.

We set the desired phase of the kicking leg at the arrival time as *φ*_d_, leading to an optimization equation:

(13) $$\begin{array}{l} n^{*},T^{*}=\underset{n,T}{\arg\min}{\left(\frac{T_{r}-nT}{T}-\varphi_{d}\right)}^{2}+\alpha_{p}\Delta T^{2}\\ S.T.\{\begin{array}{cc} \begin{array}{c} \underline{T}\leq T\leq \bar{T}\\ \bar{v}T\eta \leq \bar{L}\\ n\in \mathbb{N}\\ nT\leq T_{r} \end{array} & \begin{array}{c} \left(a\right)\\ \left(b\right)\\ \left(c\right)\\ \left(d\right) \end{array} \end{array} \end{array}$$

where Δ*T* represents the difference between the optimized and actual values of the gait cycle, and *α*_p_ is the corresponding weight. Constraint (13a) stipulates that $\underline{T}$ and $\bar{T}$ are the lower and upper limits of the cycle, respectively. Constraint (13b) involves $\bar{v}$ and $\bar{L}$, which are the maximum velocity and stride limits, respectively. This expresses the stride length constraint when the leg is in the stance state. Constraints (13c,d) represent integer constraints. Considering velocity and distance errors, *φ*_d_ is chosen to be one-ninth of the swing state in the experiment.

To make the optimization problem satisfy the standard form of mixed-integer quadratic programming [35], we transform the variable *T* to frequency, *f*, and remove the constant term. The optimization problem can then be rewritten as follows:

(14) $$\begin{array}{l} n^{*},f^{*}=\underset{n,f}{\arg\min}J=\left[\begin{array}{cc} n & f \end{array}\right]\left[\begin{array}{cc} 1 & -T_{r}\\ -T_{r} & T_{r}^{2}+\alpha_{p} \end{array}\right]\left[\begin{array}{c} n\\ f \end{array}\right]+2\left[\begin{array}{cc} \varphi_{d} & -\varphi_{d}T_{r} \end{array}-\alpha_{p}f_{\mathrm{act}}\right]\left[\begin{array}{c} n\\ f \end{array}\right]\\ S.T.\begin{array}{c} \left[\begin{array}{c} \underline{f}\\ \bar{v}\eta \\ -\infty \\ f_{\mathrm{act}}+\Delta f_{\mathrm{th}} \end{array}\right]\leq \left[\begin{array}{cc} 0 & 1\\ 0 & \bar{L}\\ 1 & -T_{r}\\ 0 & 1 \end{array}\right]\left[\begin{array}{c} n\\ f \end{array}\right]\leq \left[\begin{array}{c} \bar{f}\\ +\infty \\ 0\\ f_{\mathrm{act}}-\Delta f_{\mathrm{th}} \end{array}\right]\\ n\in \mathbb{Z} \end{array} \end{array}$$

where *f*_act_ represents the current actual gait frequency. To prevent the obtained optimal frequency from oscillating, an additional threshold, *J*_th_, is introduced. The cycle value is used only when J* < *J*_last_ − *J*_th_. It is crucial to note that upon altering the cycle *T*, beyond merely resetting *φ* to zero, the phase offsets, *φ*_Ao_ and *φ*_Bo_, require corresponding adjustments, as outlined in Table 2.

## 4. Experiments

### 4.1. Penalty Shot

**Figure 11:**
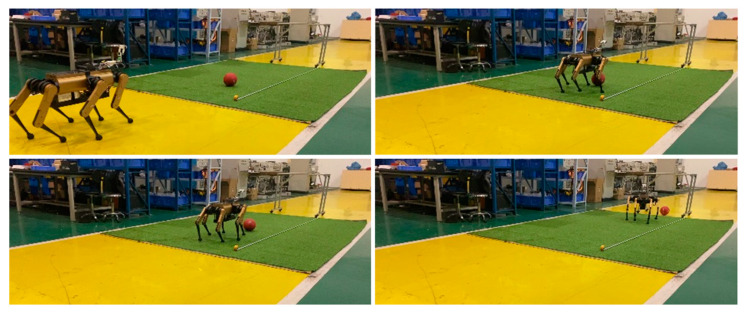
Penalty shot: Robot executes penalty shots at fixed point.

The robot was positioned 6 m directly in front of the goal that measures 1 m in width. The ball was placed 3 m directly in front of the goal. The robot, initially stationary, moved toward the ball at a speed of 0.8 m/s. When the ball entered the zone for the kicking of the selected leg, the robot performed a kicking action, as shown in Figure 11. In a series of 12 repeated experiments, the ball hit the goal five times. The remaining seven balls missed the target, with five missing entirely and two hitting the posts. The primary reason for the lack of precision in the penalty shot was an error in ball recognition. This is attributed to significant fluctuations in the camera’s depth data, causing a discrepancy between the fitted ball center and its actual position. As shown in Figure 1, the camera, attached to the robot body via two serial servos and a 3D-printed plastic cantilever beam, has low rigidity. This results in the camera shaking during the robot’s movement, further deteriorating the accuracy of the external parameters and image quality. As demonstrated in Video Part 1.2, an error in the ball’s vertical position can cause the robot to step on the ball, while a substantial horizontal deviation can turn the ball into a screwball with an unpredictable trajectory. Additionally, one of the 12 experiments encountered a sporadic hardware malfunction, where the servos failed to enable correctly, causing the robot to acquire an incorrect ball position.

### 4.2. Cycle Planning Experiment

**Figure 12:**
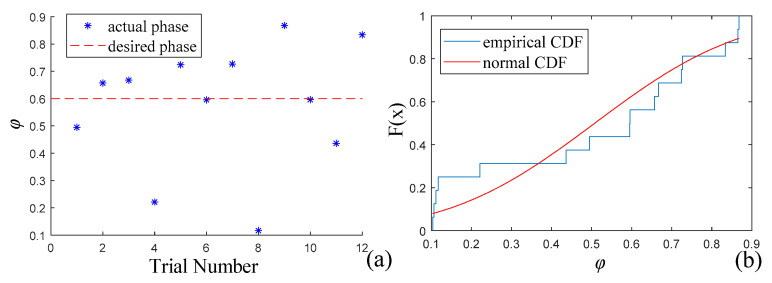
(**a**) Phase when the selected leg enters the pre-kick state in 12 experiments. (**b**) Cumulative distribution function.

In a series of 12 consecutive experiments, the robot started approximately 3 m from the ball. It dynamically adjusted its gait cycle while approaching the ball, as described in Section 3.4. The objective was for the optimally chosen kicking leg to be in the early stage of the swing state when the ball entered the zone for kicking. In these experiments, *φ*_d_ was set to 0.60, corresponding to one-ninth of the swing state. The phase of the selected kicking leg upon entering the pre-kick state was recorded in a log file, as shown in Figure 12a, with a mean value of *μ* = 0.5577 and a standard deviation of *σ* = 0.2878. The Kolmogorov–Smirnov method was used to test whether the phase distribution conformed to the normal distribution N(*μ*, *σ*^2^). The null hypothesis that the phase distribution of the 12 experiments followed this normal distribution resulted in *p* = 0.5995 (*p*-value of the test), which was significantly greater than the significance threshold of 0.05. For a more intuitive understanding of the sample regularity, the empirical cumulative distribution function and cumulative distribution function of the normal distribution are plotted in Figure 12b, showing a good fit between the sample and theoretical values. Therefore, the experimental results conformed to the hypothesized normal distribution. This indicates a clear improvement in the phase of the selected leg upon entering the pre-kick state through cycle planning (without planning, the phase was uniformly distributed between 0 and 1). The discrepancy between the experimental phase’s mean and the set target value is primarily attributed to velocity fluctuations and the limitations of cycle planning. Velocity fluctuation refers to the variation in body speed caused by the dynamic cycle adjustment, which affects the timing of the leg’s entry into the pre-kick state. The planning limitations pertain to the maximum allowable adjustment magnitude for each touchdown event. Setting the limit too low restricts the effectiveness of the cycle adjustments, whereas setting it too high can cause further disturbances to the robot body, leading to speed fluctuations.

**Figure 13:**
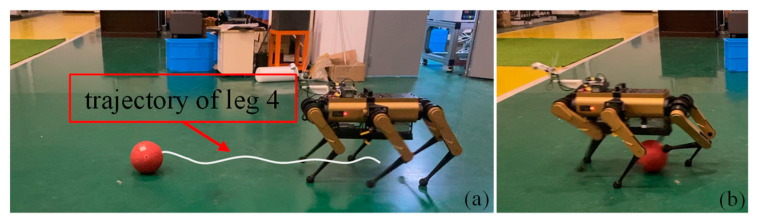
(**a**) Actual tiptoe trajectory of the kicking leg under the influence of cycle adjustment. (**b**) Back leg completes the shot when the front leg misses the ball.

According to the video recording of the first experiment, as illustrated in Figure 13a, the robot incrementally increased the stride length of its fourth leg while maintaining a uniform approach toward the ball. This adjustment results from a gradual enlargement of the gait cycle, facilitating the phase of the chosen leg to be close to the desired phase upon transitioning to the pre-kick state. The adjustment process is described in Part 2.1 of Supplementary Video S1. The robot exhibited slight oscillations when all legs were in contact with the ground, which was attributable to discontinuities in the gait cycle. Future studies will investigate the continuous modulation of the gait cycle, whereby minor adjustments to the cycle are made at 1 ms intervals. Within the 12 trials, one instance of failure was noted, as detailed in Part 2.2 of Supplementary Video S1. This failure occurred when the ball was within the kickable zone of the robot’s two front legs; however, neither leg was in a phase allowing for a kick. By the time the phase allowed either leg to kick, the ball reached the rear half of the robot, exceeding the kickable zone of the front legs. Despite the robot managing the execution of a “blind kick” with its rear left leg (Leg 1), as shown in Figure 13b, we classified this trial as unsuccessful.

### 4.3. Dynamic Dribble

**Figure 14:**
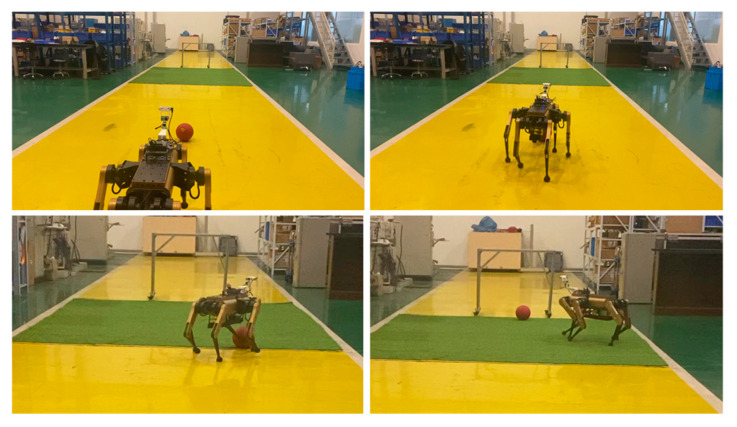
Dribbling process: robot dribbles and shoots from a long distance.

The robot was positioned 8 m directly in front of the goal, with the soccer ball positioned 1 m in front of it. From a stationary position, the robot advanced toward the ball at a speed of 0.8 m/s. It initiated dribbling to guide the ball into the goal, as illustrated in Figure 14. Owing to variations in the kick, the ball does not follow a straight path while being dribbled. However, the robot adjusts its posture and the trajectory of the kicking leg’s tiptoe to ensure that the ball is kicked into the goal. The entire process is presented in Part 3 of Supplementary Video S1.

## 5. Discussion and Conclusions

Although this algorithm was implemented on a hexapod robot platform, its theoretical principles are equally applicable to quadrupedal robots. This is because all kicking operations are performed during the swing phase of the leg, and assigning additional tasks to a swing leg has a relatively minor impact on the robot’s overall balance. However, quadrupedal robots using a trot gait enter an underactuated state, making it more challenging to maintain body balance. Therefore, although the algorithm can be conveniently transferred in theory, considering that we have not yet implemented it on a quadruped platform, its actual effectiveness still needs further verification.

The proposed algorithm allows for kicking with all six legs. However, owing to the robot’s body obstructing the camera’s field of view, most kicking actions were executed by legs 3 and 4. Legs 2 and 5 only occasionally managed to successfully kick the ball. In future studies, we plan to investigate the coordination of multiple robots. This will involve sharing the ball’s position among teammates, enabling players to execute kicks without direct visual observation.

The dynamic properties of the ball are too complex to be accurately represented using a simple spring–damper model. Therefore, this study did not consider the dynamic interaction between the ball and the tiptoe of the kicking leg; instead, the velocity of the kicking leg’s tiptoe was directly specified.

By utilizing a motion planner, which includes an optimization-based cycle-planning algorithm designed to enhance dynamic behavior, we developed a legged robot capable of performing dynamic soccer skills. A series of experiments, including penalty shots, cycle adjustment, and dynamic dribbling, validated the effectiveness of our approach. However, the current limitation is the low success rate of penalty shots. We believe that the instability of the camera mounting and fluctuations in depth data are key factors contributing to the low success rate of penalty shots. In future work, we will first focus on improving the accuracy of soccer ball-position detection. In addition to adopting a more stable mounting method, we will attempt to increase point cloud data used for sphere fitting, compare the detected circle diameter with the physical diameter of the soccer ball to verify distance measurements, and enhance data filtering. Only with more accurate detection precision can we improve the robot’s various ball-handling performances.

Past research on dynamic mobile manipulation has been limited by the high hardware costs and the complexity of simultaneously estimating the states of the robot and objects while optimizing movement and manipulation. This challenge is even greater in dynamic scenarios. Our study demonstrates that legged robots, in addition to being used in inspection scenarios, can also effectively operate in dynamic control environments requiring simultaneous movement and manipulation. And their interaction with physical objects is not limited to carrying objects or using legged manipulators.

## Figures and Tables

**Table 1:** Slicing of the workspace for each leg.

*i-*th Leg	Zone in Body Coordinate System (z = −0.305)
0	(x + 0.33)^2^ + (y + 0.053)^2^ < 0.433, −0.0012 > y > −0.304
1	(x + 0.33)^2^ + (y − 0.053)^2^ < 0.433, 0.0012 < y < 0.304
2	x^2^ + (y − 0.19025)^2^ < 0.433, 0.0685 < y < 0.511
3	(x − 0.33)^2^ + (y − 0.053)^2^ < 0.433, 0.0012 < y < 0.304
4	(x − 0.33)^2^ + (y + 0.053)^2^ < 0.433, −0.0012 > y > −0.304
5	x^2^ + (y + 0.19025)^2^ < 0.433, −0.0685 > y > −0.511

**Table 2:** Adjustment of phase offsets.

/	A Stance &&((*φ*_Ao_ = 0, *φ* ≥ 0.5) || (*φ*_Ao_ = 0.5, *φ* < 0.5))	A Stance &&((*φ*_Ao_ = 0, *φ* < 0.5) || (*φ*_Ao_ = 0.5, *φ* ≥ 0.5))	A Swing
B stance	*φ*_Ao_ = 0.5, *φ*_Bo_ = 0	*φ*_Ao_ = 0, *φ*_Bo_ = 0.5	*φ*_Ao_ = 0, *φ*_Bo_ = 0.5
B swing	*φ*_Ao_ = 0.5, *φ*_Bo_ = 0	*φ*_Ao_ = 0.5, *φ*_Bo_ = 0	/

## Data Availability

The data were generated using the proposed model.

## Supplementary Materials

The following supporting information can be downloaded at https://www.mdpi.com/article/10.3390/s24186070/s1. Video S1.
